# 4-Benzyl-3,5-dimethyl-1*H*-pyrazole

**DOI:** 10.1107/S1600536811045405

**Published:** 2011-11-05

**Authors:** Su-Qing Wang, Cheng Kong

**Affiliations:** a MicroScale Science Institute, Department of Chemistry and Chemical Engineering, Weifang University, Weifang 261061, People’s Republic of China; b MicroScale Science Institute, Weifang University, Weifang 261061, People’s Republic of China

## Abstract

In the title mol­ecule, C_12_H_14_N_2_, the dihedral angle between the pyrazole and phenyl ring mean planes is 78.65 (19)°. In the crystal, mol­ecules are linked by N—H⋯N hydrogen bonds into chains along [010].

## Related literature

For the pharmacological activity of pyrazole derivatives, see: Adnan & Tarek (2004 ▶); Ashraf *et al.* (2003 ▶). For a related structure, see: Wang & Jian (2010 ▶). For standard bond-length data, see: Allen *et al.* (1987 ▶).
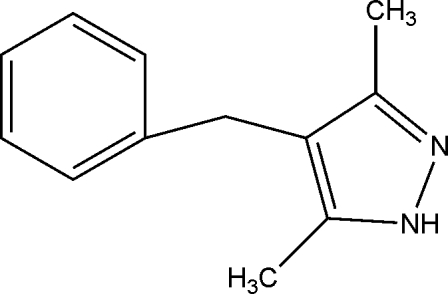


## Experimental

### 

#### Crystal data

C_12_H_14_N_2_
*M* *_r_* = 186.25
Monoclinic, 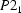

*a* = 6.2303 (6) Å
*b* = 5.5941 (5) Å
*c* = 15.1364 (15) Å
β = 97.049 (1)°
*V* = 523.56 (9) Å^3^
*Z* = 2
Mo *K*α radiation
μ = 0.07 mm^−1^
*T* = 298 K
0.48 × 0.32 × 0.15 mm

#### Data collection

Bruker SMART CCD diffractometer
Absorption correction: multi-scan (*SADABS*; Sheldrick, 1996 ▶) *T*_min_ = 0.967, *T*_max_ = 0.989
2666 measured reflections
1023 independent reflections
756 reflections with *I* > 2σ(*I*)
*R*_int_ = 0.043

#### Refinement

*R*[*F* ^2^ > 2σ(*F* ^2^)] = 0.045
*wR*(*F* ^2^) = 0.107
*S* = 0.95
1023 reflections
127 parameters
1 restraint
H-atom parameters constrained
Δρ_max_ = 0.11 e Å^−3^
Δρ_min_ = −0.13 e Å^−3^

### 

Data collection: *SMART* (Bruker, 1997 ▶); cell refinement: *SAINT* (Bruker, 1997 ▶); data reduction: *SAINT*; program(s) used to solve structure: *SHELXS97* (Sheldrick, 2008 ▶); program(s) used to refine structure: *SHELXL97* (Sheldrick, 2008 ▶); molecular graphics: *SHELXTL* (Sheldrick, 2008 ▶); software used to prepare material for publication: *SHELXTL*.

## Supplementary Material

Crystal structure: contains datablock(s) global, I. DOI: 10.1107/S1600536811045405/lh5342sup1.cif

Structure factors: contains datablock(s) I. DOI: 10.1107/S1600536811045405/lh5342Isup2.hkl

Supplementary material file. DOI: 10.1107/S1600536811045405/lh5342Isup3.cml

Additional supplementary materials:  crystallographic information; 3D view; checkCIF report

## Figures and Tables

**Table 1:** Hydrogen-bond geometry (Å, °)

*D*—H⋯*A*	*D*—H	H⋯*A*	*D*⋯*A*	*D*—H⋯*A*
N2—H2⋯N1^i^	0.86	2.09	2.946 (4)	170

Symmetry code: (i) 
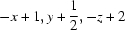
.

**Fig. 1.**
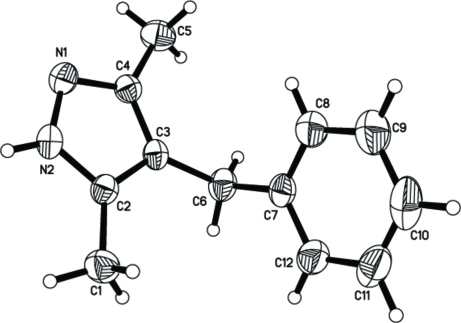
The molecular structure, drawn with 30% probability ellipsoids and spheres of arbritrary size for the H atoms.

## supplementary crystallographic information

### Comment

Pyrazole and its derivatives are an important class of N-heterocyclic compounds because they exhibit a broad spectrum of pharmacological activities such as antifungal (Adnan & Tarek, 2004), antitumor and antiangiogenic activities (Ashraf *et al.*, 2003). As part of our research based on pyrazole derivatives and there complexes, the crystal structure of aquabis(3,5-dimethylpyrazolyl) copper(II) sulfate hydrate has been determined (Wang & Jian, 2010). As part of this ongoing search for new pyrazole compounds, the title compound was synthesized and its crystal structure is reported herein. In the title molecule (Fig. 1), bond lengths (Allen *et al.*, 1987) and angles fall in normal ranges. The dihedral angle between the pyrazole ring (N1/N2/C2-C4) and the phenyl ring (C7-C11) is 78.65 (19)°. In the crystal, molecules are linked by N—H···N hydrogen bonds into one-dimensional chains along [010].

### Experimental

A mixture of 3-benzylpentane-2,4-dione (7.03 g, 0.037 mol) and hydrazine hydrate (2.20 g, 0.044 mol) was stirred with ice water for 4h. The reaction mixture was poured into ice water (100 ml) and the aqueous layer was extracted with ether. After being dried over anhydrous potassium carbonate, the organic layer was evaporated and the residue was purified. Single crystals were obtained by evaporation of an petroleum ether solution of (I) at room temperature over a period of several days.

### Refinement

In the absense of significant anomalous dispersion effects Friedel pairs were merged. The H atoms were placed in calculated positions (C—H = 0.93–0.97 Å and N—H = 0.86Å), and refined as riding with *U*_iso_(H) = 1.2*U*_eq_(C, N) or 1.5U_eq_(methyl C).

### Crystal data

**Table:**

C_12_H_14_N_2_	*F*(000) = 200
*M**_r_* = 186.25	*D*_x_ = 1.181 Mg m^−^^3^
Monoclinic, *P*2_1_	Mo *K*α radiation, λ = 0.71073 Å
Hall symbol: P 2yb	Cell parameters from 648 reflections
*a* = 6.2303 (6) Å	θ = 2.7–20.1°
*b* = 5.5941 (5) Å	µ = 0.07 mm^−^^1^
*c* = 15.1364 (15) Å	*T* = 298 K
β = 97.049 (1)°	Block, colorless
*V* = 523.56 (9) Å^3^	0.48 × 0.32 × 0.15 mm
*Z* = 2	

### Data collection

**Table:**

Bruker SMART CCD diffractometer	1023 independent reflections
Radiation source: fine-focus sealed tube	756 reflections with *I* > 2σ(*I*)
graphite	*R*_int_ = 0.043
φ and ω scans	θ_max_ = 25.0°, θ_min_ = 2.7°
Absorption correction: multi-scan (*SADABS*; Sheldrick, 1996)	*h* = −6→7
*T*_min_ = 0.967, *T*_max_ = 0.989	*k* = −6→6
2666 measured reflections	*l* = −16→18

### Refinement

**Table:**

Refinement on *F*^2^	Primary atom site location: structure-invariant direct methods
Least-squares matrix: full	Secondary atom site location: difference Fourier map
*R*[*F*^2^ > 2σ(*F*^2^)] = 0.045	Hydrogen site location: inferred from neighbouring sites
*wR*(*F*^2^) = 0.107	H-atom parameters constrained
*S* = 0.95	*w* = 1/[σ^2^(*F*_o_^2^) + (0.0568*P*)^2^] where *P* = (*F*_o_^2^ + 2*F*_c_^2^)/3
1023 reflections	(Δ/σ)_max_ < 0.001
127 parameters	Δρ_max_ = 0.11 e Å^−^^3^
1 restraint	Δρ_min_ = −0.13 e Å^−^^3^

### Special details

**Table:**

Geometry. All e.s.d.'s (except the e.s.d. in the dihedral angle between two l.s. planes) are estimated using the full covariance matrix. The cell e.s.d.'s are taken into account individually in the estimation of e.s.d.'s in distances, angles and torsion angles; correlations between e.s.d.'s in cell parameters are only used when they are defined by crystal symmetry. An approximate (isotropic) treatment of cell e.s.d.'s is used for estimating e.s.d.'s involving l.s. planes.
Refinement. Refinement of *F*^2^ against ALL reflections. The weighted *R*-factor *wR* and goodness of fit *S* are based on *F*^2^, conventional *R*-factors *R* are based on *F*, with *F* set to zero for negative *F*^2^. The threshold expression of *F*^2^ > σ(*F*^2^) is used only for calculating *R*-factors(gt) *etc*. and is not relevant to the choice of reflections for refinement. *R*-factors based on *F*^2^ are statistically about twice as large as those based on *F*, and *R*- factors based on ALL data will be even larger.

### Fractional atomic coordinates and isotropic or equivalent isotropic displacement parameters (Å^2^)

**Table:**

	*x*	*y*	*z*	*U*_iso_*/*U*_eq_	
N1	0.4939 (4)	0.1998 (6)	0.91543 (15)	0.0558 (7)	
N2	0.6284 (4)	0.3826 (6)	0.94362 (13)	0.0536 (7)	
H2	0.6092	0.4729	0.9880	0.064*	
C1	0.9603 (5)	0.5915 (7)	0.91602 (19)	0.0672 (10)	
H1A	0.9315	0.7234	0.8758	0.101*	
H1B	1.0990	0.5241	0.9091	0.101*	
H1C	0.9604	0.6464	0.9761	0.101*	
C2	0.7922 (4)	0.4085 (6)	0.89629 (17)	0.0471 (7)	
C3	0.7654 (4)	0.2339 (6)	0.83141 (16)	0.0451 (7)	
C4	0.5795 (5)	0.1098 (6)	0.84614 (18)	0.0479 (8)	
C5	0.4757 (5)	−0.0964 (8)	0.7964 (2)	0.0703 (10)	
H5A	0.3904	−0.1838	0.8341	0.106*	
H5B	0.5851	−0.1993	0.7779	0.106*	
H5C	0.3842	−0.0398	0.7450	0.106*	
C6	0.9109 (5)	0.1927 (8)	0.76133 (18)	0.0600 (9)	
H6A	0.8811	0.0356	0.7356	0.072*	
H6B	1.0597	0.1934	0.7891	0.072*	
C7	0.8863 (5)	0.3750 (7)	0.68827 (18)	0.0548 (8)	
C8	0.6936 (6)	0.4084 (9)	0.6353 (2)	0.0737 (10)	
H8	0.5753	0.3144	0.6446	0.088*	
C9	0.6716 (7)	0.5754 (9)	0.5699 (2)	0.0910 (14)	
H9	0.5393	0.5939	0.5348	0.109*	
C10	0.8423 (9)	0.7162 (9)	0.5551 (2)	0.0953 (14)	
H10	0.8268	0.8309	0.5104	0.114*	
C11	1.0345 (8)	0.6876 (9)	0.6063 (3)	0.0900 (14)	
H11	1.1517	0.7829	0.5967	0.108*	
C12	1.0566 (6)	0.5183 (8)	0.6721 (2)	0.0726 (11)	
H12	1.1897	0.4998	0.7067	0.087*	

### Atomic displacement parameters (Å^2^)

**Table:**

	*U*^11^	*U*^22^	*U*^33^	*U*^12^	*U*^13^	*U*^23^
N1	0.0603 (15)	0.0557 (18)	0.0530 (14)	−0.0014 (16)	0.0135 (12)	0.0051 (16)
N2	0.0644 (16)	0.0562 (18)	0.0419 (12)	0.0048 (19)	0.0135 (11)	0.0001 (15)
C1	0.074 (2)	0.060 (2)	0.066 (2)	−0.012 (2)	0.0049 (17)	−0.0028 (19)
C2	0.0515 (17)	0.0453 (18)	0.0449 (14)	0.0028 (18)	0.0073 (12)	0.0069 (17)
C3	0.0527 (17)	0.0435 (19)	0.0393 (14)	0.0099 (17)	0.0063 (13)	0.0033 (15)
C4	0.0495 (17)	0.0473 (19)	0.0462 (16)	0.0033 (16)	0.0036 (14)	0.0014 (16)
C5	0.070 (2)	0.059 (2)	0.081 (2)	−0.005 (2)	0.0025 (17)	−0.007 (2)
C6	0.0623 (19)	0.064 (2)	0.0563 (17)	0.011 (2)	0.0169 (15)	−0.003 (2)
C7	0.064 (2)	0.057 (2)	0.0461 (15)	0.005 (2)	0.0173 (15)	−0.0083 (19)
C8	0.080 (2)	0.081 (3)	0.0602 (19)	−0.009 (3)	0.0101 (18)	0.005 (3)
C9	0.106 (3)	0.099 (4)	0.068 (2)	0.001 (3)	0.007 (2)	0.019 (3)
C10	0.158 (4)	0.073 (3)	0.060 (2)	−0.002 (4)	0.031 (3)	0.008 (2)
C11	0.124 (4)	0.081 (3)	0.074 (2)	−0.033 (3)	0.046 (3)	−0.016 (3)
C12	0.076 (2)	0.080 (3)	0.065 (2)	−0.012 (2)	0.0212 (19)	−0.009 (2)

### Geometric parameters (Å, °)

**Table:**

N1—C4	1.332 (3)	C6—C7	1.498 (5)
N1—N2	1.357 (4)	C6—H6A	0.9700
N2—C2	1.325 (3)	C6—H6B	0.9700
N2—H2	0.8600	C7—C8	1.372 (5)
C1—C2	1.469 (4)	C7—C12	1.375 (5)
C1—H1A	0.9600	C8—C9	1.357 (5)
C1—H1B	0.9600	C8—H8	0.9300
C1—H1C	0.9600	C9—C10	1.364 (6)
C2—C3	1.381 (5)	C9—H9	0.9300
C3—C4	1.392 (4)	C10—C11	1.353 (6)
C3—C6	1.495 (3)	C10—H10	0.9300
C4—C5	1.482 (5)	C11—C12	1.370 (6)
C5—H5A	0.9600	C11—H11	0.9300
C5—H5B	0.9600	C12—H12	0.9300
C5—H5C	0.9600		
			
C4—N1—N2	103.9 (2)	C3—C6—C7	113.7 (3)
C2—N2—N1	113.5 (2)	C3—C6—H6A	108.8
C2—N2—H2	123.2	C7—C6—H6A	108.8
N1—N2—H2	123.2	C3—C6—H6B	108.8
C2—C1—H1A	109.5	C7—C6—H6B	108.8
C2—C1—H1B	109.5	H6A—C6—H6B	107.7
H1A—C1—H1B	109.5	C8—C7—C12	117.1 (4)
C2—C1—H1C	109.5	C8—C7—C6	121.8 (3)
H1A—C1—H1C	109.5	C12—C7—C6	121.1 (3)
H1B—C1—H1C	109.5	C9—C8—C7	121.6 (4)
N2—C2—C3	105.9 (3)	C9—C8—H8	119.2
N2—C2—C1	122.9 (3)	C7—C8—H8	119.2
C3—C2—C1	131.2 (3)	C8—C9—C10	120.4 (4)
C2—C3—C4	105.6 (2)	C8—C9—H9	119.8
C2—C3—C6	125.7 (3)	C10—C9—H9	119.8
C4—C3—C6	128.7 (3)	C11—C10—C9	119.4 (4)
N1—C4—C3	111.0 (3)	C11—C10—H10	120.3
N1—C4—C5	120.1 (3)	C9—C10—H10	120.3
C3—C4—C5	128.8 (3)	C10—C11—C12	120.1 (4)
C4—C5—H5A	109.5	C10—C11—H11	120.0
C4—C5—H5B	109.5	C12—C11—H11	120.0
H5A—C5—H5B	109.5	C11—C12—C7	121.4 (4)
C4—C5—H5C	109.5	C11—C12—H12	119.3
H5A—C5—H5C	109.5	C7—C12—H12	119.3
H5B—C5—H5C	109.5		
			
C4—N1—N2—C2	−0.6 (3)	C2—C3—C6—C7	−74.5 (4)
N1—N2—C2—C3	0.7 (3)	C4—C3—C6—C7	105.8 (4)
N1—N2—C2—C1	−178.7 (3)	C3—C6—C7—C8	−60.6 (5)
N2—C2—C3—C4	−0.5 (3)	C3—C6—C7—C12	118.7 (3)
C1—C2—C3—C4	178.8 (3)	C12—C7—C8—C9	0.0 (5)
N2—C2—C3—C6	179.8 (3)	C6—C7—C8—C9	179.3 (3)
C1—C2—C3—C6	−1.0 (5)	C7—C8—C9—C10	−0.2 (6)
N2—N1—C4—C3	0.2 (3)	C8—C9—C10—C11	0.2 (7)
N2—N1—C4—C5	−179.9 (3)	C9—C10—C11—C12	0.0 (7)
C2—C3—C4—N1	0.1 (3)	C10—C11—C12—C7	−0.3 (6)
C6—C3—C4—N1	179.9 (3)	C8—C7—C12—C11	0.2 (5)
C2—C3—C4—C5	−179.8 (3)	C6—C7—C12—C11	−179.1 (3)
C6—C3—C4—C5	0.0 (5)		

### Hydrogen-bond geometry (Å, °)

**Table:**

*D*—H···*A*	*D*—H	H···*A*	*D*···*A*	*D*—H···*A*
N2—H2···N1^i^	0.86	2.09	2.946 (4)	170

Symmetry codes: (i) −*x*+1, *y*+1/2, −*z*+2.
